# Safety of an Enhanced Recovery Pathway for Patients Undergoing Open Hepatic Resection

**DOI:** 10.1371/journal.pone.0150782

**Published:** 2016-03-07

**Authors:** Clancy J. Clark, Shahzad M. Ali, Victor Zaydfudim, Adam K. Jacob, David M. Nagorney

**Affiliations:** 1 Department of General Surgery, Wake Forest Baptist Health, Winston-Salem, North Carolina, United States of America; 2 Division of Gastroenterologic and General Surgery, Mayo Clinic, Rochester, Minnesota, United States of America; 3 Department of General Surgery, University of Virginia, Charlottesville, Virginia, United States of America; 4 Department of Anesthesiology, Mayo Clinic, Rochester, Minnesota, United States of America; ISMETT-UPMC Italy/ University of Catania, ITALY

## Abstract

**Background:**

Enhanced recovery pathways (ERP) have not been widely implemented for hepatic surgery. The aim of this study was to evaluate the safety of an ERP for patients undergoing open hepatic resection.

**Methods:**

A single-surgeon, retrospective observational cohort study was performed comparing the clinical outcomes of patients undergoing open hepatic resection treated before and after implementation of an ERP. Morbidity, mortality, and length of hospital stay (LOS) were compared between pre-ERP and ERP groups.

**Results:**

126 patients (pre-ERP n = 73, ERP n = 53) were identified for the study. Patient characteristics and operative details were similar between groups. Overall complication rate was similar between pre-ERP and ERP groups (37% vs. 28%, p = 0.343). Before and after pathway implementation, the median LOS was similar, 5 (IQR 4–7) vs. 5 (IQR 4–6) days, p = 0.708. After adjusting for age, type of liver resection, and ASA, the ERP group had no increased risk of major complication (OR 0.38, 95% CI 0.14–1.02, p = 0.055) or LOS greater than 5 days (OR 1.21, 95% CI 0.56–2.62, p = 0.627).

**Conclusions:**

Routine use of a multimodal ERP is safe and is not associated with increased postoperative morbidity after open hepatic resection.

## Introduction

Clinical care pathways have emerged over the last decade in the management of patients who have undergone major gastrointestinal operations. These pathways have the goal of decreasing variability, enhancing quality of care, improving postoperative outcomes, and decreasing health care delivery costs.[1] Such treatment strategies were pioneered in colorectal surgery in the 1990’s and frequently referred to as an enhanced recovery pathway (ERP), fast-track pathway, clinical or critical pathway, accelerated rehabilitation, or standardized care protocol.[2] For example, implementation of an ERP in colorectal surgery patients at our institution resulted in decreased length of stay and decreased postoperative morbidity for patients who have undergone partial colectomy.[3] Similar experiences with major abdominal operations have been reported both in the United States and abroad.[4–14]

A key concept of an ERP is a protocol-driven multidisciplinary team approach and involvement of healthcare providers throughout a patient’s care pathway including at time of pre-operative evaluation, as well as, in the induction area, operating room, post-anesthesia care unit, and postoperative surgical care unit. Success of an ERP requires is protocol-driven integration of team members and team care.

The key components of an ERP include: 1) pre-operative education regarding the operation and expected recovery; 2) omission of bowel preparation; 3) anesthetic and analgesic regimen that minimizes postoperative sedation and systemic opiates; 4) immediate postoperative discontinuation of nasogastric tube; 5) early feeding; 6) enforced ambulation on the day of surgery, and 7) early removal of bladder catheter.[3] Other useful features of an ERP are educational discharge planning, prewritten order pathway, prokinetic medications, neutral intra-operative and post-operative fluid balance, antiembolic stockings, a standardized approach to laboratory studies, specific discharge criteria and education regarding these criteria early in postoperative period, breathing exercises, and an organized post-operative physical therapy program.[1]

Application of an ERP for hepatic resection has been very limited likely due to concerns regarding the safety of routine use of spinal anesthesia, non-steroidal anti-inflammatory drugs, and venous thromboembolism prophylaxis. Several studies outside the United States indicate such protocols are safe and effective in reducing length of stay.[15–23] Anecdotal evidence supported safe implementation of colorectal ERP protocols among patients undergoing combined hepatic resection and colectomy at our institution. This experience prompted implementation of an ERP for patients undergoing hepatic resection alone. The aim of the current study was to evaluate the safety of an enhanced recovery pathway following open hepatic resection compared to the standard postoperative management of historical controls after hepatic resection.

## Methods

### Study Cohort

This single-surgeon, retrospective observational cohort study was approved by the Mayo Clinic Institutional Review Board. Written consent was waived for this study. All data was de-identified for analysis. Between January 2010 and December 2010, individual components of the ERP were piloted. Specifically, preoperative and intraoperative anesthetics and fluid management protocols were standardized by the Department of Anesthesiology. Education and training of nursing staff, pharmacists, discharge planners, and social workers, was also performed during this period. Importantly, the ERP was introduced on a hospital ward where a similar pathway had been implemented previously by the colorectal surgery at the Mayo Clinic.[3] In January 2011, a multimodal ERP was implemented for all adult patients undergoing hepatic resection by a single hepatobiliary surgeon (D.M.N.) at the Methodist Hospital, Mayo Clinic, Rochester. Patients with polycystic liver disease requiring major hepatic resection or hilar cholangiocarcinoma requiring major hepatic resection and biliary-enteric anastomosis were excluded from the current study. In addition, patients undergoing laparoscopic hepatic resections, planned multivisceral resections, radiofrequency ablation without resection, or emergent operations were also excluded from the study. Patients with chronic renal insufficiency were included in the study because the protocol permits changes in medication selection and dosing to adjust for impaired glomerular filtration rate. Patients with contraindications to neuraxial analgesia were also enrolled. Excluding the transition interval during 2010, the current study compared patients during a period from January 1, 2009 to December 31, 2009 (pre-ERP group) to patients in a period from January 1, 2011 to June 30, 2011 (ERP group). Analysis was performed on an intent-to-treat basis.

### Enhanced Recovery Protocol

All patients scheduled to undergo elective open liver resection were enrolled in the ERP of January 1, 2011. Components of the ERP are summarized in **Table 1**. Components of the ERP were based on our experience with an ERP in colorectal surgery and review of the current literature.[1,3] Key aspects of the ERP were standardized anesthetic with premedication, intrathecal spinal analgesia, postoperative oral narcotics on day 0; minimization of time interval with no oral intake; early mobilization; minimization of intraoperative and postoperative intravenous fluids; removal of naso- or oro- gastric tube at the time of emergence from anesthesia with early feeding, and standardized postoperative care. Intrathecal spinal analgesia was not performed prior to introduction of the ERP. Subcostal or midline incision was used for all patients. Perihepatic drainage was not a component of the ERP. Intraperitoneal surgical drains were used selectively after major hepatectomy when concern for bile leak was present. Following the operation, patients were transferred to the post-anesthesia care unit (PACU) and routinely transferred to a standard hospital ward for postoperative care. Transfer from the operating room or PACU to the intensive care unit (ICU) was determined on a case-by-case basis depending on patient co-morbidities or peri-operative hemodynamic stability. Discharge criteria were not modified with implementation of the ERP. Patients were discharged if functionally recovered from the operation. Functionally recovered was defined as afebrile for 24 hours; normal or decreasing serum bilirubin; good pain control on oral analgesics only; absence of intravenous fluids, tolerance of diet, and ambulation with minimal assistance.[16]

**Table 1:** Enhanced Recovery Pathway after Hepatic Resection.

**Preoperative**	**1**	Preoperative education regarding the operation and expected recovery.
	**2**	Omission of bowel preparation.
	**3**	Preoperative medication with gabapentin, celecoxib, and acetaminophen.
**Intraoperative**		
	**4**	Standardized anesthetic with intrathecal analgesia.
**Postoperative**		
	**5**	Routine postoperative antiemetics.
	**6**	Discontinuation of nasogastric tube before leaving the operating room.
	**7**	Ketorolac/Ibuprofen and acetaminophen with supplemental oral narcotic, starting night of operation.
	**8**	General diet with clear liquid oral nutritional supplement, starting night of operation.
	**9**	Enforced ambulation and in chair for all meals, starting night of operation.
	**10**	Foley catheter removal, day after operation.
	**11**	Minimal intravenous fluids and saline lock, day after operation.
	**12**	Venous thromboembolism prophylaxis.
	**13**	Standardized bowel regimen.
	**14**	Standardized laboratory tests.

### Data Acquisition and Definitions

Medical records were reviewed to extract demographic information, preoperative weight, history of liver disease, comorbidities, smoking history, prior abdominal operation, American Society of Anesthesiology (ASA) classification, indication for operation, and pathology findings. Perioperative data included type of operation, extent of hepatic resection, vascular resection and reconstruction, total intraoperative fluids, estimated blood loss, reoperation rate, readmission rate, immediate postoperative transfer to the intensive care unit, and postoperative 30-day morbidity and mortality. Length of hospital stay was defined as the number of days in the hospital excluding the day of the operation. Major liver resection was defined as four or more segments removed.[24] Severity of complications was categorized using an updated version of the T92 score referred to as the “Accordion Classification”.[25] The following categories were used: 1) Mild–minor invasive procedure (e.g. nasogastric tube placement), minor medical therapy (e.g. diuretic); 2) Moderate–antibiotic therapy, transfusion, parenteral nutrition; 3) Severe–endoscopic or interventional radiology procedure, reoperation, organ failure, and 4) Death. Major complication was defined as complication severity two or greater.

### Statistical Analysis

Statistical analyses were performed using SAS^®^ 9.1.3 (SAS Institute Inc., Cary, NC, USA). All data were collected and managed using RedCap.[26] Univariate comparison of pre-ERP and ERP was performed using Chi-square and Fischer exact test for categorical variables (as appropriate) and Wilcoxon rank-sum test for continuous variables. Continuous data was reported as median with interquartile range (IQR) and categorical data as count and percent. Logistic regression was used to evaluate the influence of covariates on postoperative major complication rate and length of hospital stay. Significance was defined as p-value ≤ 0.05.

## Results

One hundred and twenty-six patients (median age 62, male 58%) were identified for the study (pre-ERP n = 73, ERP n = 53). The main indication for open hepatic resection was colorectal metastases (33%, n = 42). No patients underwent minimally-invasive techniques. Underlying liver disease was uncommon (hepatitis B, n = 1; hepatitis C, n = 3; primary sclerosing cholangitis, n = 2). Three patients (2.4%) had a history of cirrhosis. Comparison of clinical characteristics between pre-ERP and ERP groups is summarized in **Table 2**. Age, sex, American Society of Anesthesiologists (ASA) score, comorbidities, alcohol abuse, and tobacco use were not significantly different between pre-ERP and ERP groups (all p > 0.1).

**Table 2:** Comparison of clinical characteristics between Pre- ERP vs. ERP groups.

Variable	Overall (n = 126)	Pre-ERP (n = 73)	ERP (n = 53)	p-value
Age, median (IQR)	62 (50–72)	65 (51–73)	59 (47–68)	0.114
Male Sex, No. (%)	58 (46.0)	36 (49.3)	22 (41.5)	0.470
ASA, No. (%)				
I	4 (3.2)	3 (4.1)	1 (1.9)	0.709
II	71 (56.4)	42 (57.5)	29 (54.7)	
III	51 (40.5)	28 (38.4)	23 (43.4)	
Comorbidity, No. (%)				
Chronic Renal Failure	5 (4.0)	3 (4.1)	2 (3.8)	>0.999
Diabetes Mellitus	24 (19.1)	13 (17.8)	11 (20.8)	0.819
Coronary Artery Disease	8 (6.4)	6 (8.2)	2 (3.8)	0.466
Obesity, BMI > 30, No. (%)	46 (36.5)	24 (32.9)	22 (41.5)	0.353
COPD	6 (4.8)	4 (5.5)	2 (3.8)	>0.999
Alcohol Abuse, No. (%)	15 (11.9)	10 (13.7)	5 (9.4)	0.582
History of Tobacco Use, No. (%)	68 (54.0)	38 (52.1)	30 (56.6)	0.718
Prior Abdominal Operation, No. (%)	96 (76.2)	53 (72.6)	43 (81.1)	0.297
Prior Hepatic Resection, No. (%)	16 (12.7)	7 (9.6)	9 (17.0)	0.281
Liver Disease, No. (%)				
Hepatitis B	1 (0.8)	1 (1.4)	0 (0)	>0.999
Hepatitis C	3 (2.4)	1 (1.4)	2 (3.8)	0.572
Cirrhosis	3 (2.4)	2 (2.7)	1 (1.9)	>0.999
Primary Sclerosing Cholangitis	2 (1.6)	1 (1.4)	1 (1.9)	>0.999
Indication for Operation, No. (%)				
Colorectal Metastases	42 (33.3)	22 (30.1)	20 (37.7)	0.317
Cholangiocarcinoma	20 (15.9)	14 (19.2)	6 (11.3)	
Hepatocellular Carcinoma	18 (14.3)	8 (11.0)	10 (18.9)	
Benign Primary Liver Tumor	16 (12.7)	12 (16.4)	4 (7.6)	
Neuroendocrine Metastases	13 (10.3)	8 (11.0)	5 (9.4)	
Gallbladder Carcinoma	8 (3.4)	3 (4.1)	5 (9.4)	
Other	9 (7.1)*	6 (8.2)	3 (5.7)	

MELD, Model for End Stage Liver Disease; COPD, Chronic Obstructive Pulmonary Disease; IQR, Interquartile Range; ASA, American Society of Anesthesiologists; ERP, enhanced recovery pathway

* Other included hemangioma n = 6, intrahepatic biliary benign stricture n = 1, indeterminate intrahepatic fibrous tumor n = 1, and primary hepatic neuroendocrine tumor n = 1.

Major hepatectomy (4 or more segments) was performed in 37% (n = 47) of patients. Only five patients (4%) required vascular repair or reconstruction. Comparison of operative details between pre-ERP and ERP groups is summarized in **Table 3**. Type of procedure, vascular repair or reconstruction, use of radiofrequency ablation, estimated blood loss, and need for intraoperative blood transfusion were not significantly different between pre-ERP and ERP groups (all p > 0.100).

**Table 3:** Comparison of operative details between Pre-ERP vs. ERP groups.

Variable	Overall (n = 126)	Pre-ERP (n = 73)	ERP (n = 53)	p-value
Major Hepatic Liver Resection, No. (%)	47 (37.3)	30 (41.1)	17 (32.1)	0.353
Procedure, No. (%)				
Subsegmentectomy	22 (17.5)	13 (17.8)	9 (17.0)	0.861
Multiple Subsegmentectomies	30 (23.8)	16 (21.9)	14 (26.4)	
Single Segmentectomy	9 (7.1)	5 (6.9)	4 (7.6)	
Multiple Segments	24 (19.1)	12 (16.4)	12 (22.6)	
Hepatectomy	29 (23.0)	19 (26.0)	10 (18.9)	
Extended Hepatectomy	12 (9.5)	8 (11.0)	4 (7.6)	
Vascular Resection or Repair, No. (%)	5 (4.0)	3 (4.1)	2 (3.8)	>0.999
Radiofrequency Ablation, No. (%)	18 (14.3)	10 (13.7)	8 (15.1)	>0.999
Estimated Blood Loss, ml, median (IQR)	400 (150–650)	400 (150–600)	350 (200–700)	0.812
Intraoperative Blood Transfusion, No. (%)	17 (13.5)	11 (15.1)	6 (11.3)	0.606
Intraoperative IV Fluids, ml, median (IQR)	3000 (2250–4000)	3000 (2000–4000)	3500 (2500–4100)	0.044
Transfer to ICU Immediately After Operation, No. (%)	8 (6.4)	4 (5.5)	4 (7.6)	0.720

IQR, Interquartile Range; ICU, Intensive Care Unit; ERP, Enhanced Recovery Pathway

To determine the safety of the enhanced recovery pathway, overall complication and major complication rates were compared between pre-ERP and ERP groups. Clinical outcomes are summarized in **Table 4**. Overall, four patients (3.2%) required reoperation within 30 days for intra-abdominal bleeding (n = 3) and fascial dehiscence (n = 1). Unplanned transfer to the intensive care unit for postoperative complication was not different between the ERP vs. pre-ERP group, (7.6% vs. 13.7%, p = 0.392). Overall complication rate did not differ between the ERP vs. pre-ERP group, (28.3% vs. 37.0%, p = 0.343). Major complication rate was also similar between the ERP vs. pre-ERP group, (13.2% vs. 26.0%, p = 0.118). After adjusting for age, type of resection, and ASA, patients in the ERP group had no increased risk of major complication (OR 0.38, 95% CI 0.14–1.02, p = 0.055) compared to pre-ERP cohort. Comparison of specific postoperative complications between pre-ERP and ERP groups is summarized in **Table 5**.

**Table 4:** Comparison of postoperative outcomes between Pre- ERP vs. ERP groups.

Variable	Overall (n = 126)	Pre-ERP (n = 73)	ERP (n = 53)	p-value
Length of Stay, days, median (IQR)	5 (4–7)	5 (4–7)	5 (4–6)	0.708
Postoperative Blood Transfusion, No. (%)	4 (3.2)	2 (2.7)	2 (3.8)	>0.999
Percutaneous Drainage, No. (%)	5 (4.0)	4 (5.5)	1 (1.9)	0.397
Reoperation, No. (%)	4 (3.2)	2 (2.7)	2 (3.8)	>0.999
Unplanned Postoperative ICU Transfer, No. (%)	14 (11.1)	10 (13.7)	4 (7.6)	0.392
Readmission, 90-day, No. (%)	4 (3.2)	2 (2.7)	2 (3.8)	>0.999
Any Complication, No. (%)	42 (33.3)	27 (37.0)	15 (28.3)	0.343
Complication Severity Score, No. (%)				
None	84 (66.7)	46 (63.0)	38 (71.7)	0.476
Mild	16 (12.7)	8 (11.0)	8 (15.1)	
Moderate	16 (12.7)	11 (15.1)	5 (9.4)	
Severe	9 (7.1)	7 (9.6)	2 (3.8)	
Death	1 (0.8)	1 (1.4)	0 (0)	

IQR, Interquartile Range; ICU, intensive care unit; ERP, Enhanced Recovery Pathway

**Table 5:** Comparison of specific postoperative complications between Pre- ERP vs. ERP groups. No significant differences between individual complications were identified (all p-values > 0.1).

Complication*	Overall (n = 126)	Pre-ERP (n = 73)	ERP (n = 53)
Acute Renal Failure	5 (4.0)	3 (4.1)	2 (3.8)
Bile Leak	6 (4.8)	5 (6.9)	1 (1.9)
Deep Venous Thrombosis	4 (3.2)	3 (4.1)	1 (1.9)
Pulmonary Embolism	3 (2.4)	2 (2.7)	1 (1.9)
Intra-abdominal Bleeding	3 (2.4)	1 (1.4)	2 (3.8)
Hepatic Insufficiency	3 (2.4)	3 (4.1)	0 (0)
Ileus	8 (6.4)	5 (6.9)	3 (5.7)
Pneumonia	6 (4.8)	5 (6.9)	1 (1.9)
Respiratory failure requiring intubation	4 (3.2)	4 (5.5)	0 (0)
Septic Shock	1 (0.8)	1 (1.4)	0 (0)
Superficial Skin Infection	2 (1.6)	0 (0)	2 (3.8)
Cardiac Dysrhythmia	5 (4.0)	3 (4.1)	2 (3.8)

*Other complications not listed include severe postoperative delirium (n = 1), chylous ascites (n = 1), and failure to thrive requiring total parental nutrition (n = 1). Complications due to myocardial infection, bacteremia, or intra-abdominal abscess unrelated to bile leak were not identified during the study period

Median length of hospital stay was the same for pre-ERP and ERP groups, 5 days (4–7) and 5 days (4–6), respectively, p = 0.708. After adjusting for age, type of resection, and ASA, there was no difference in risk of length of hospitalization exceeding 5 days between the ERP vs. pre-ERP groups (OR 1.21, 95% CI 0.56–2.62, p = 0.627). Rates of 90-day post-operative re-admission did not differ between pre-ERP and ERP groups, 2.7% and 3.8%, respectively, p > 0.999. Indication for readmission was as follows: biloma (n = 1), hepatic insufficiency (n = 1), postoperative nausea and vomiting (n = 1), and hepatic vein outflow obstruction (n = 1). Sub-group analysis of major hepatectomy patients did not demonstrate significant differences in LOS (p = 0.350) before and after introduction of ERP. However, for major hepatectomy patients, the minimum LOS for ERP patients was two days compared with four days in historical controls.

## Discussion

Multiple studies have demonstrated that coordinated multidisciplinary clinical care pathways can decrease variability and improve outcomes after major gastrointestinal operations.[2,4–6,8–11,13,14,16] Lemmens et al., in a systematic review, outlined that clinical pathways can decrease length of stay without adversely impacting postoperative complication rates, re-admission rates, or mortality.[1] However, data to support safety of fast-track and ERP for hepatic resection is limited.[21,22] The current study sought to investigate the safety of a multimodal ERP for patients after hepatic resection. Comparison of pre-ERP and ERP patients demonstrated no increase in overall and major complication rates. Despite significant involvement of providers throughout the patient’s hospital course and a regimented care pathway, length of stay and percent re-admitted were similar between pre-ERP and ERP groups.

While the current study does not demonstrate a clear benefit of an ERP over standard care, our study does suggest a tendency toward a lower number of patients in the ERP group who encountered complications. Similarly, fewer patients required transfer to the ICU for complications in the ERP group. Specifically, in the ERP group, the number of patients developing respiratory and venous complications (pulmonary emboli, deep vein thrombosis) was slightly lower possibly due to early mobilization and routine use of intrathecal analgesia.

In an international survey of hepatobiliary centers by Wong-Lun-Hing et al., 28% (45 of 161) of medical centers had experience with fast-track perioperative care for patients after hepatic resection.[20] In this same study, surgeon-reported median length of stay was five days in North America and seven days in Europe for patients treated with open hepatic resection. Consistent with other hepatobiliary centers in the United States, our median length of stay was five days. Previous studies report a two to three day decrease of length of stay with introduction of an ERP.[16,19,23] The decrease in length of stay reported in these studies may be a reflection of changes in surgical dogma among general surgery practices or changes in patient expectations and cultural shifts in perioperative care rather than the direct result of individual components of the ERP.[16,23] Nursing engagement before and after surgery may have also played a role in minimal change in LOS with introduction of the ERP. Future studies will need to investigate the specific clinical and financial benefit of each component of the ERP. With introduction of laparoscopic and less invasive techniques, length of stay for hepatic resection will likely go below the apparent threshold of five days for open hepatic resection.

The current study has several limitations. It was a retrospective comparative cohort study limited to a single surgeon’s practice. The study is limited to open procedures and does not address the potential benefit of laparoscopic approaches. In our experience, laparoscopic liver resection significantly reduces length of hospital stay by two to four days. However, major hepatic resection represents a small proportion of laparoscopic procedures at our institution and elsewhere.[19,27] Additionally, although providers throughout the patient’s care pathway received training on the hepatic ERP, we did not measure provider or patient compliance. In a prospective study design, we would be able to measure compliance to an ERP as well as evaluate the potential benefits particularly related to improved pain control, quality-of-life, and patient satisfaction. Since this was a study focused on the safety of an ERP for patients with hepatic resection, we also did not evaluate the potential financial benefit of ERP. Lastly, the current study was performed in a high-volume hepatobiliary center with a senior hepatobiliary surgeon. The potential benefits of a hepatic ERP may be more evident in other hospital settings.

In conclusion, implementation of an ERP for open hepatic resection is safe and does not increase perioperative morbidity or mortality. While prior studies of enhanced recovery pathways for major gastrointestinal procedures indicate a reduction in length of stay, length of stay was unaffected by implementation of an ERP for patients undergoing hepatic resection.

## References

[1] Lemmens L, van Zelm R, Borel Rinkes I, van Hillegersberg R, Kerkkamp H. Clinical and organizational content of clinical pathways for digestive surgery: a systematic review. Dig Surg. 2009;26: 91–9. 10.1159/000206142 19252405

[2] Bradshaw BG, Liu SS, Thirlby RC. Standardized perioperative care protocols and reduced length of stay after colon surgery. J Am Coll Surg. 1998;186: 501–6. Available: http://www.ncbi.nlm.nih.gov/pubmed/9583689. 9583689 10.1016/s1072-7515(98)00078-7

[3] Tsikitis VL, Holubar SD, Dozois EJ, Cima RR, Pemberton JH, Larson DW. Advantages of fast-track recovery after laparoscopic right hemicolectomy for colon cancer. Surg Endosc. 2010;24: 1911–6. 10.1007/s00464-009-0871-y 20108142

[4] Porter GA, Pisters PW, Mansyur C, Bisanz A, Reyna K, Stanford P, et al Cost and utilization impact of a clinical pathway for patients undergoing pancreaticoduodenectomy. Ann Surg Oncol. 2000;7: 484–9. Available: http://www.ncbi.nlm.nih.gov/pubmed/10947015. 10947015 10.1007/s10434-000-0484-0

[5] Balcom JH, Rattner DW, Warshaw a L, Chang Y, Fernandez-del Castillo C. Ten-year experience with 733 pancreatic resections: changing indications, older patients, and decreasing length of hospitalization. Arch Surg. 2001;136: 391–8. Available: http://www.ncbi.nlm.nih.gov/pubmed/11296108. 11296108 10.1001/archsurg.136.4.391

[6] Delaney CP, Fazio VW, Senagore AJ, Robinson B, Halverson AL, Remzi FH. “Fast track” postoperative management protocol for patients with high co-morbidity undergoing complex abdominal and pelvic colorectal surgery. Br J Surg. 2001;88: 1533–8. 10.1046/j.0007-1323.2001.01905.x 11683754

[7] Delaney CP, Zutshi M, Senagore AJ, Remzi FH, Hammel J, Fazio VW. Prospective, randomized, controlled trial between a pathway of controlled rehabilitation with early ambulation and diet and traditional postoperative care after laparotomy and intestinal resection. Dis Colon Rectum. 2003;46: 851–9. 10.1097/01.DCR.0000075209.09377.00 12847356

[8] Melbert RB, Kimmins MH, Isler JT, Billingham RP, Lawton D, Salvadalena G, et al Use of a critical pathway for colon resections. J Gastrointest Surg. 2002;6: 745–52. Available: http://www.ncbi.nlm.nih.gov/pubmed/12399065. 12399065 10.1016/s1091-255x(02)00038-0

[9] Stephen AE, Berger DL. Shortened length of stay and hospital cost reduction with implementation of an accelerated clinical care pathway after elective colon resection. Surgery. 2003;133: 277–82. 10.1067/msy.2003.19 12660639

[10] Raue W, Haase O, Junghans T, Scharfenberg M, Müller JM, Schwenk W. “Fast-track” multimodal rehabilitation program improves outcome after laparoscopic sigmoidectomy: a controlled prospective evaluation. Surg Endosc. 2004;18: 1463–8. 10.1007/s00464-003-9238-y 15791370

[11] Basse L, Thorbøl JE, Løssl K, Kehlet H. Colonic surgery with accelerated rehabilitation or conventional care. Dis Colon Rectum. 2004;47: 271–7; discussion 277–8. 10.1007/s10350-003-0055-0 14991487

[12] Kennedy EP, Grenda TR, Sauter PK, Rosato EL, Chojnacki K a, Rosato FE, et al Implementation of a critical pathway for distal pancreatectomy at an academic institution. J Gastrointest Surg. 2009;13: 938–44. 10.1007/s11605-009-0803-0 19190968

[13] Kennedy EP, Rosato EL, Sauter PK, Rosenberg LM, Doria C, Marino IR, et al Initiation of a critical pathway for pancreaticoduodenectomy at an academic institution—the first step in multidisciplinary team building. J Am Coll Surg. 2007;204: 917–23; discussion 923–4. 10.1016/j.jamcollsurg.2007.01.057 17481510

[14] Hirao M, Tsujinaka T, Takeno A, Fujitani K, Kurata M. Patient-controlled dietary schedule improves clinical outcome after gastrectomy for gastric cancer. World J Surg. 2005;29: 853–7. 10.1007/s00268-005-7760-x 15951932

[15] MacKay G, O’Dwyer PJ. Early discharge following liver resection for colorectal metastases. Scott Med J. 2008;53: 22–4. Available: http://www.ncbi.nlm.nih.gov/pubmed/18549066. 10.1258/rsmsmj.53.2.22 18549066

[16] van Dam RM, Hendry PO, Coolsen MME, Bemelmans MHA, Lassen K, Revhaug A, et al Initial experience with a multimodal enhanced recovery programme in patients undergoing liver resection. Br J Surg. 2008;95: 969–75. 10.1002/bjs.6227 18618897

[17] Koea JB, Young Y, Gunn K. Fast track liver resection: the effect of a comprehensive care package and analgesia with single dose intrathecal morphine with gabapentin or continuous epidural analgesia. HPB Surg. 2009;2009: 271986 10.1155/2009/271986 20029637 PMC2796218

[18] Schultz N a, Larsen PN, Klarskov B, Plum LM, Frederiksen HJ, Christensen BM, et al Evaluation of a fast-track programme for patients undergoing liver resection. Br J Surg. 2013;100: 138–43. 10.1002/bjs.8996 23165484

[19] Stoot JH, van Dam RM, Busch OR, van Hillegersberg R, De Boer M, Olde Damink SWM, et al The effect of a multimodal fast-track programme on outcomes in laparoscopic liver surgery: a multicentre pilot study. HPB (Oxford). 2009;11: 140–4. 10.1111/j.1477-2574.2009.00025.x 19590638 PMC2697877

[20] Wong-Lun-Hing EM, Lodewick TM, Stoot JHMB, Bemelmans MHA, Olde Damink SWM, Dejong CHC, et al A survey in the hepatopancreatobiliary community on ways to enhance patient recovery. HPB (Oxford). 2012;14: 818–27. 10.1111/j.1477-2574.2012.00546.x 23134183 PMC3521910

[21] Hall TC, Dennison AR, Bilku DK, Metcalfe MS, Garcea G. Enhanced recovery programmes in hepatobiliary and pancreatic surgery: a systematic review. Ann R Coll Surg Engl. 2012;94: 318–26. 10.1308/003588412X13171221592410 22943226 PMC3954372

[22] Spelt L, Ansari D, Sturesson C, Tingstedt B, Andersson R. Fast-track programmes for hepatopancreatic resections: where do we stand? HPB (Oxford). 2011;13: 833–8. 10.1111/j.1477-2574.2011.00391.x 22081917 PMC3244621

[23] Lin D-X, Li X, Ye Q-W, Lin F, Li L-L, Zhang Q-Y. Implementation of a Fast-Track Clinical Pathway Decreases Postoperative Length of Stay and Hospital Charges for Liver Resection. Cell Biochem Biophys. 2011; 413–419. 10.1007/s12013-011-9203-7 21556940 PMC3210369

[24] Strasberg SM. Nomenclature of hepatic anatomy and resections: a review of the Brisbane 2000 system. J Hepatobiliary Pancreat Surg. 2005;12: 351–5. 10.1007/s00534-005-0999-7 16258801

[25] Strasberg SM, Linehan DC, Hawkins WG. The accordion severity grading system of surgical complications. Ann Surg. 2009;250: 177–86. 10.1097/SLA.0b013e3181afde41 19638919

[26] Harris PA, Taylor R, Thielke R, Payne J, Gonzalez N, Conde JG. Research electronic data capture (REDCap)—a metadata-driven methodology and workflow process for providing translational research informatics support. J Biomed Inform. 2009;42: 377–81. 10.1016/j.jbi.2008.08.010 18929686 PMC2700030

[27] Nguyen KT, Marsh JW, Tsung A, Steel JJL, Gamblin TC, Geller D a. Comparative Benefits of Laparoscopic vs Open Hepatic Resection: A Critical Appraisal. Arch Surg. 2011;146: 348–56. 10.1001/archsurg.2010.248 21079109

